# Supplementary material for: Smokeless tobacco use and oral potentially malignant disorders among people living with HIV (PLHIV) in Pune, India: Implications for oral cancer screening in PLHIV
Source: PLoS One. 2022 Jul 5;17(7):e0270876. doi: 10.1371/journal.pone.0270876 (PMC9255739; doi:10.1371/journal.pone.0270876)
Supplement: S1 File — (DOCX) [file pone.0270876.s001.docx]

**Supporting Information**

**Part A: Summary of the method used to obtain corrected prevalence and prevalence ratios (PRs)**

**Part B: Supplementary graphs and tables**

1. **S1 Fig. Corrected prevalence of OPMDs under the entire range of plausible sensitivity and specificity parameters assuming non-differential misclassification of suspected OPMDs (a. For the overall study population; b. For PLHIV; c. For the HIV uninfected group)**
2. **S2a Fig. Corrected adjusted prevalence ratios for OPMDs comparing PLHIV to HIV-uninfected individuals under different sensitivity and specificity parameters assuming non-differential misclassification of suspected OPMDs**
3. **S2b Fig. Corrected adjusted prevalence ratios for OPMDs among PLHIV comparing current smokeless tobacco users to never smokeless tobacco users under different sensitivity and specificity parameters assuming non-differential misclassification of suspected OPMDs**
4. **S1 Table: Prevalence ratios for suspected OPMDs among participants recruited in an mHealth-based oral cancer screening study in Pune, India when sex is included as a covariate**
5. **S2 Table: Adjusted prevalence ratios for suspected OPMDs among participants recruited in an mHealth-based oral cancer screening study in Pune, India comparing models where Oral HPV status was excluded as a covariate to models where Oral HPV values were imputed**
6. **S3 Fig**: **Adjusted prevalence ratios for suspected OPMDs among current smokeless tobacco users by HIV status and duration of use, when imputed oral HPV status is included as a covariate**
