# Supplementary material for: Smokeless tobacco use and oral potentially malignant disorders among people living with HIV (PLHIV) in Pune, India: Implications for oral cancer screening in PLHIV
Source: PLoS One. 2022 Jul 5;17(7):e0270876. doi: 10.1371/journal.pone.0270876 (PMC9255739; doi:10.1371/journal.pone.0270876)

# **Supplementary files**

# **PART A: Summary of the method used to obtain corrected prevalence and prevalence ratios (PRs)**

Since only 46% of participants complied with follow-up procedures, we were unable to use the positive predictive value (probability of a positive clinical diagnosis on follow-up given positive suspected OPMD) to obtain corrected PRs for misclassification of the dependent variable. Instead, we used validity parameters reported in various literature sources [sensitivity (0·25 – 0·99) and specificity (0·52 – 0·99)], comparing remote (images) to in-person diagnosis of oral conditions, to obtain corrected PRs. A range of corrected PRs [with 95% Confidence Intervals (CI)] was obtained for different sets of validity parameters representing potential estimates for the association between clinically diagnosed OPMDs and the explanatory variable.

We obtained corrected PRs using the following methodology. First, conditional on the observed frequencies, we created simulated data sets to calculate corrected frequencies using formulas by Lash et al^1^. As each clinician made an images review diagnosis independent of SLT use and HIV status knowledge, non-differential misclassification of suspected OPMD was assumed. Second, validity parameters for PLHIV and HIV uninfected individuals that did not result in negative or corrected frequencies approaching infinity, were considered plausible values. Prevalence, positive (PPVs) and negative predictive values (NPVs) were calculated for the range of plausible parameters. Using the upper limit (18.5%) of the 95% CI reported in the meta-analysis by Mello et al for prevalence of clinically diagnosed OPMDs in Asia as a threshold, we excluded validity parameters associated with prevalence>18.5% for HIV uninfected individuals. Given non-differential misclassification, the overlapping set of validity parameters between HIV uninfected and PLHIV were used in the analysis. Lastly, corrected adjusted (same covariates as multivariable model) odds ratios were estimated by incorporating validity parameters into the maximum likelihood estimation as described by Neuhaus, Lyles and Lin, and Lyles et al^2,3^. The odds ratios obtained were then transformed into corrected adjusted PRs.

1. Literature sources used to guide the range of sensitivity and specificity parameters comparing images review against in-person clinical examination

| **Author** | **Sensitivity** | **Specificity** |
| --- | --- | --- |
| Alabdullah JH et al^4^ | 0·25 – 1·00 | 0·52 – 1·00 |
| Vinayagamoorthy K et al^5^ | 0·98 – 0·99 | 0·52 – 0·72 |
| Haron N et al^6^ | 0·81 – 0·93 | 1·00 |

1. Method used to estimate the plausible range of sensitivity and specificity parameters for our given data

Using the sensitivity and specificity parameters identified from the literature sources cited above, corrected frequencies for the dependent variable (OPMD) were calculated using the formulas specified by Lash et al^1^ in the book, “Applying Quantitative Bias Analysis to Epidemiologic Data”.

|  | Observed | | Expected truth | |
| --- | --- | --- | --- | --- |
|  | X1 | X0 | X1 | X0 |
| Y+ | a | b | A | B |
| Y- | c | d | C | D |
| Total | a+c | b+d |  |  |

|  | Observed | | Expected truth | |
| --- | --- | --- | --- | --- |
|  | X1 | X0 | X1 | X0 |
| Y+ | a | b | [a - X_1Total_(1 – Sp_E1_)]/ [Se_E1_ – (1-Sp_E1_)] | [b – X_0Total_(1-Sp_E0_)]/ [Se_E0_ – (1 – Sp_E0_)] |
| Y- | c | d | X_1Tota_l - A | X_0Total_ - B |
| Total | a+c | b+d | A+C | B + D |
| Se- sensitivity parameter; Sp- Specificity parameter; X_1Total_= a + c; X_0Total_ = b + d  Assuming non-differential misclassification: Sp_E1 =_ Sp_E0_ and Se_E1_ = Se_E0_ | | | | |

| **Example**: Parameters Se = 0·25, Sp=0·90 | | | | |
| --- | --- | --- | --- | --- |
|  | Observed | | Expected truth | |
|  | HIV1 | HIV0 | X1 | X0 |
| OPMD+ | 117 | 69 | 379·3 | 37·9 |
| OPMD- | 484 | 564 | 221·7 | 595 |
| Total | 601 | 633 |  |  |

Simulated datasets were generated in which frequencies for the expected truth were calculated when sensitivity and specificity parameters were varied. Plausible sensitivity and specificity parameters for our given observed frequencies were those that did not result in expected truth frequencies that were negative or approached infinity. We also concurrently estimated the prevalence of OPMD, using the expected truth frequencies.

1. Prevalence estimates for OPMD from the metanalysis by Mello et al^7^, used to fix the upper limit of prevalence for OPMD for HIV uninfected individuals

| **Regional prevalence of oral potentially malignant disorders** | | | |
| --- | --- | --- | --- |
|  | No of studies | Prevalence | 95% CI |
| Asian populations | 5 | 10·54% | 4·60 – 18·55% |
| South America and the Caribbean | 7 | 3·93% | 2·43 – 5·77% |
| Middle East | 2 | 3·72% | 2·91 – 4·67% |
| Europe | 5 | 3·07% | 1·64 – 4·93% |
| North America | 2 | 0·11% | 0·004 – 0·37% |

Since non-differential misclassification: Sp_E1 =_ Sp_E0_ and Se_E1_ = Se_E0_ was assumed, the overlapping set of parameters for HIV uninfected individuals and PLHIV were used.

1. Method used to estimate the corrected adjusted prevalence ratios (PRs)
2. The method proposed by Lash et al and described above could only determine PRs for univariate models. To calculate a corrected adjusted PR, we first calculated the corrected adjusted odds ratios by modifying the estimation of the likelihood function, as proposed by Neuhaus, Lyles and Lin, and Lyles et al ^2,3^. The modification of the likelihood function is shown below.

$$\boldsymbol{L=}\prod_{\boldsymbol{i=1}}^{\boldsymbol{n}} \begin{aligned} {\boldsymbol{\{}\left( \mathbf{1-Sp} \right)\mathbf{Pr}\left( \mathbf{Y=0} | \mathbf{X=}\mathbf{x}_{\mathbf{i}} \right)\mathbf{+SePr}\left( \mathbf{Y=1} | \mathbf{X=}\mathbf{x}_{\mathbf{i}} \right)\boldsymbol{\}}}^{\mathbf{y}_{\mathbf{i}}^{\mathbf{*}}} \\ {\boldsymbol{\{SpPr}\left( \mathbf{Y=0} | \mathbf{X=}\mathbf{x}_{\mathbf{i}} \right)\mathbf{+}\left( \mathbf{1-Se} \right)\mathbf{Pr}\left( \mathbf{Y=1} | \mathbf{X=}\mathbf{x}_{\mathbf{i}} \right)\boldsymbol{\}}}^{\mathbf{1-y}_{\mathbf{i}}^{\mathbf{*}}} \end{aligned}\boldsymbol{*}$$

Y= {OPMD}

X= {Primary independent variable of interest and covariates}

1. The corrected adjusted odds ratios were then transformed into corrected adjusted PRs by using the formulation below:

$\{1/(1+$ $e^{-\left( \beta_{0} + \beta_{1} + \beta_{2}*a+ \beta_{3}*b + \ldots\ldots..\beta_{n}*z \right)})\} /$ $\{1/(1+e^{-\left( \beta_{0} +\beta_{2}*a+ \beta_{3}*b + \ldots\ldots.\beta_{n}*z \right)})\}$

where, $\beta_{0}$ is the log odds, $\beta_{1}$ is the log odds ratio for the variable one is interested in obtaining the corrected adjusted PR,$\beta_{3}-\beta_{n}$are log odds ratios for covariates, and *a-z* are the values that the covariates take in the observed data.

## **References**

1. Lash T, Fox M, Fink A. Disease Misclassification, Corrections with Sensitivity and Specificity: Nondifferential and Independent Errors. In: Applying Quantitative Bias Analysis to Epidemiologic Data. Springer; 2009:94-96.

2. Lyles RH, Tang L, Superak HM, et al. Validation data-based adjustments for outcome misclassification in logistic regression: an illustration. Epidemiology. 2011;22(4):589-597.

3. Shaw PA, Gustafson P, Carroll RJ, et al. STRATOS guidance document on measurement error and misclassification of variables in observational epidemiology: Part 2-More complex methods of adjustment and advanced topics. Stat Med. 2020;39(16):2232-2263.

4. Alabdullah JH, Daniel SJ. A Systematic Review on the Validity of Teledentistry. Telemed J E Health. 2018;24(8):639-48.

5. Vinayagamoorthy K, Acharya S, Kumar M, Pentapati KC, Acharya S. Efficacy of a remote screening model for oral potentially malignant disorders using a free messaging application: A diagnostic test for accuracy study. Aust J Rural Health. 2019;27(2):170-6.

6. Haron N, Zain RB, Nabillah WM, Saleh A, Kallarakkal TG, Ramanathan A, et al. Mobile Phone Imaging in Low Resource Settings for Early Detection of Oral Cancer and Concordance with Clinical Oral Examination. Telemed J E Health. 2017;23(3):192-9.

7. Mello FW, Miguel AFP, Dutra KL, et al. Prevalence of oral potentially malignant disorders: A systematic review and meta-analysis. J Oral Pathol Med. 2018;47(7):633-640.

# **PART B: Supplementary graphs and tables**

Please note that as we assume non-differential misclassification of the outcome variable (i.e., Sensitivity_PLHIV_ = Sensitivity_HIV uninfected_; Specificity_PLHIV_ = Specificity_HIV uninfected_), the main findings presented in the manuscript take into account the joint plausible sensitivity and specificity parameters. Thus, the lower limit of sensitivity is 0.39 (as this is the minimum plausible value of sensitivity for PLHIV) and lower limit of specificity is 0.96 (as this is the minimum plausible value of specificity for HIV uninfected individuals)

**S1 Fig: Corrected prevalence of OPMDs under the entire range of plausible sensitivity and specificity parameters assuming non-differential misclassification of suspected OPMDs**

1. **For the overall study population**

**
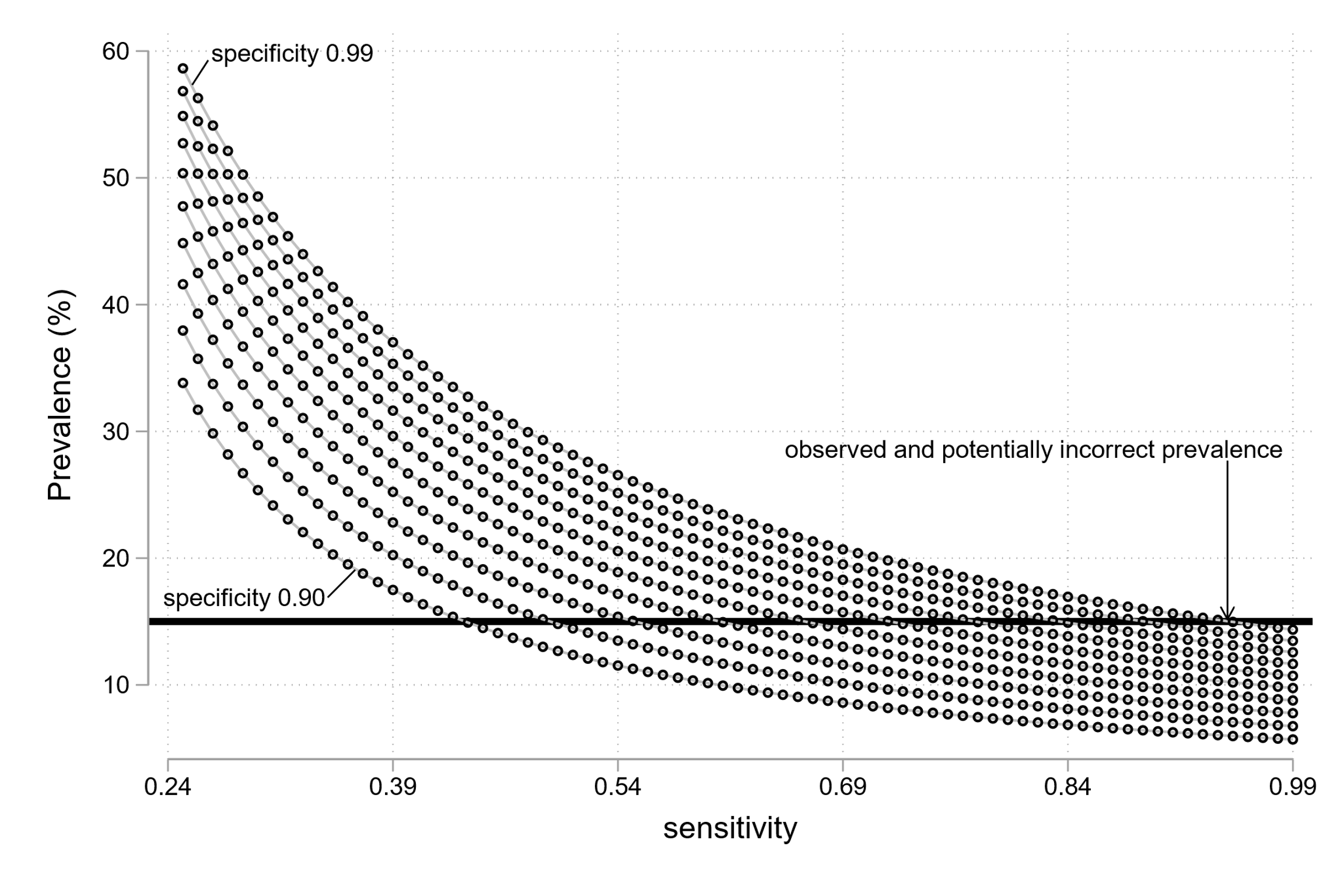
**

1. **For PLHIV**

**
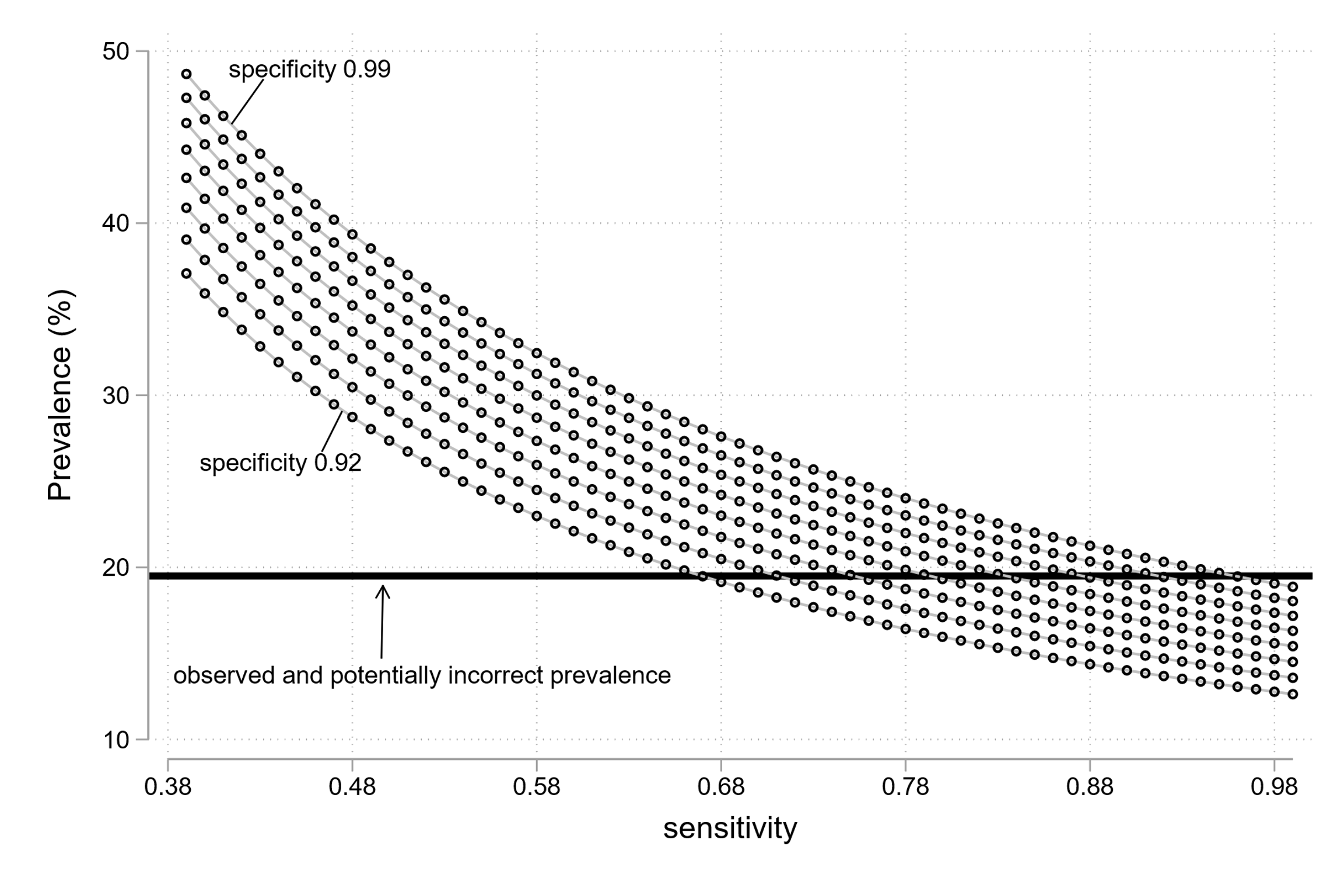
**

1. **For the HIV uninfected group**

**
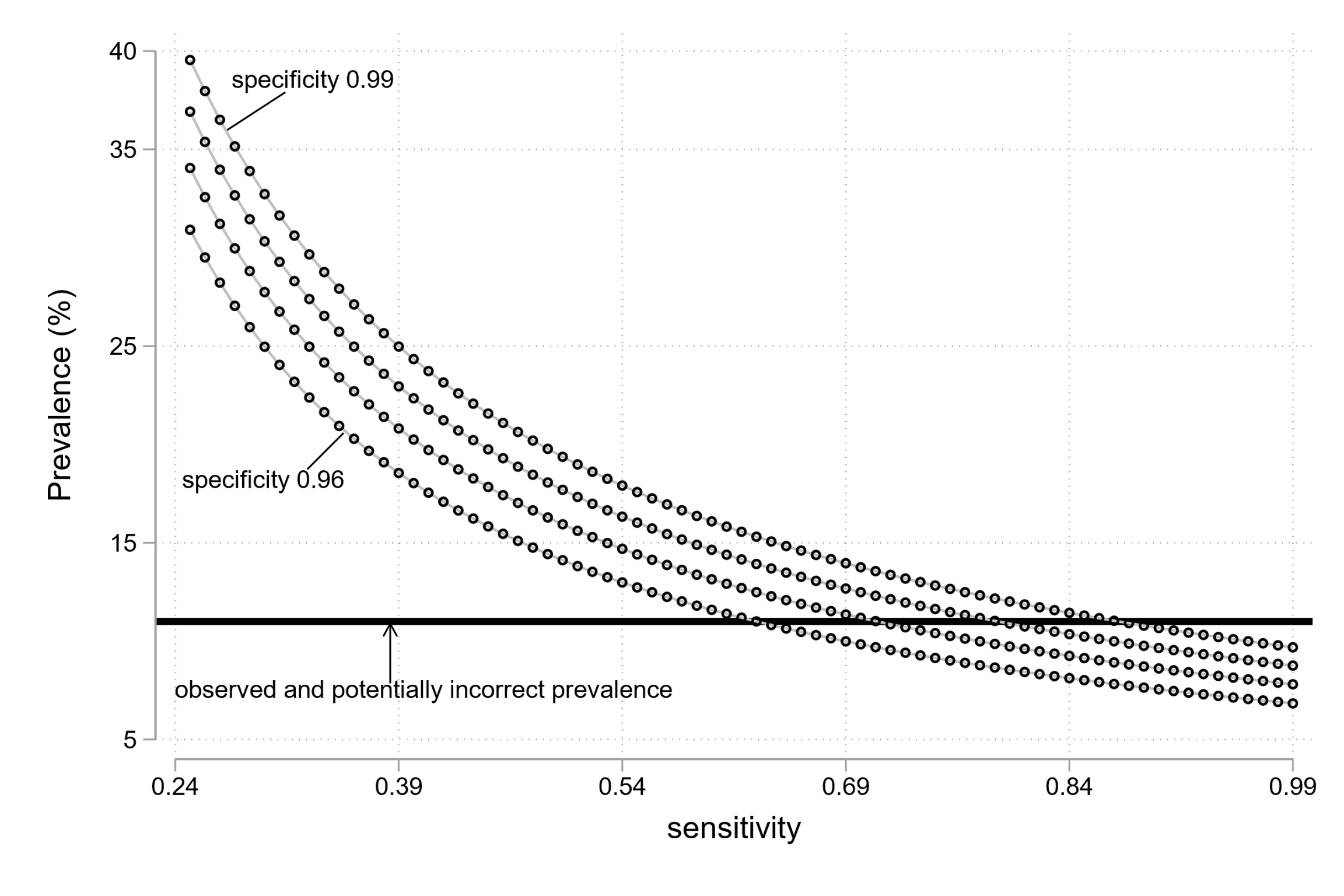
**

**S2a Fig.**

**Corrected adjusted prevalence ratios for OPMDs comparing PLHIV to HIV-uninfected individuals under different sensitivity and specificity parameters assuming non-differential misclassification of suspected OPMDs**


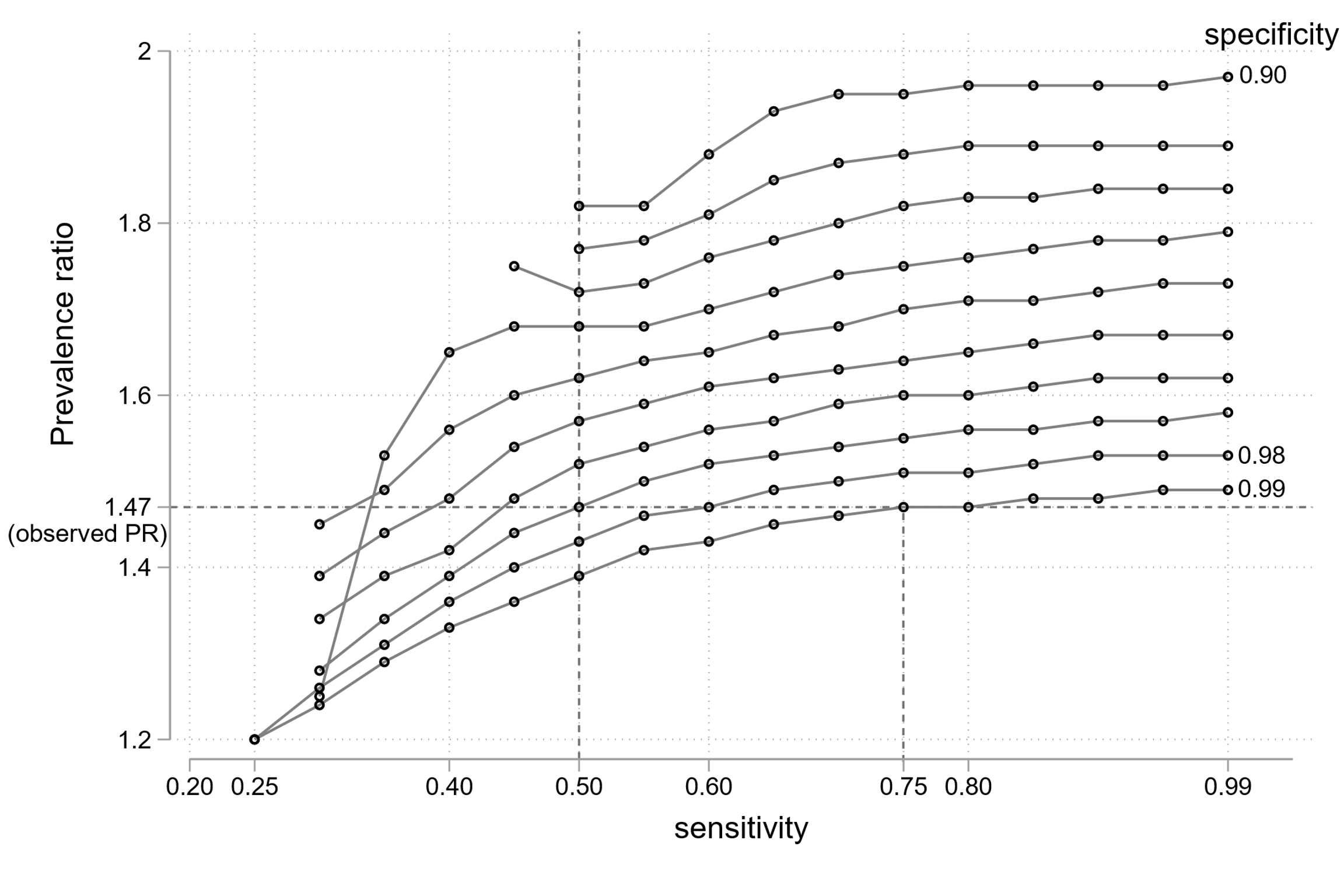


When the sensitivity≤0·60 and/or specificity ≤0·94, the models did not converge, shown as missing values in the graph.

**S2b Fig:**

**Corrected adjusted prevalence ratios for OPMDs among PLHIV comparing current smokeless tobacco users to never smokeless tobacco users under different sensitivity and specificity parameters assuming non-differential misclassification of suspected OPMDs**


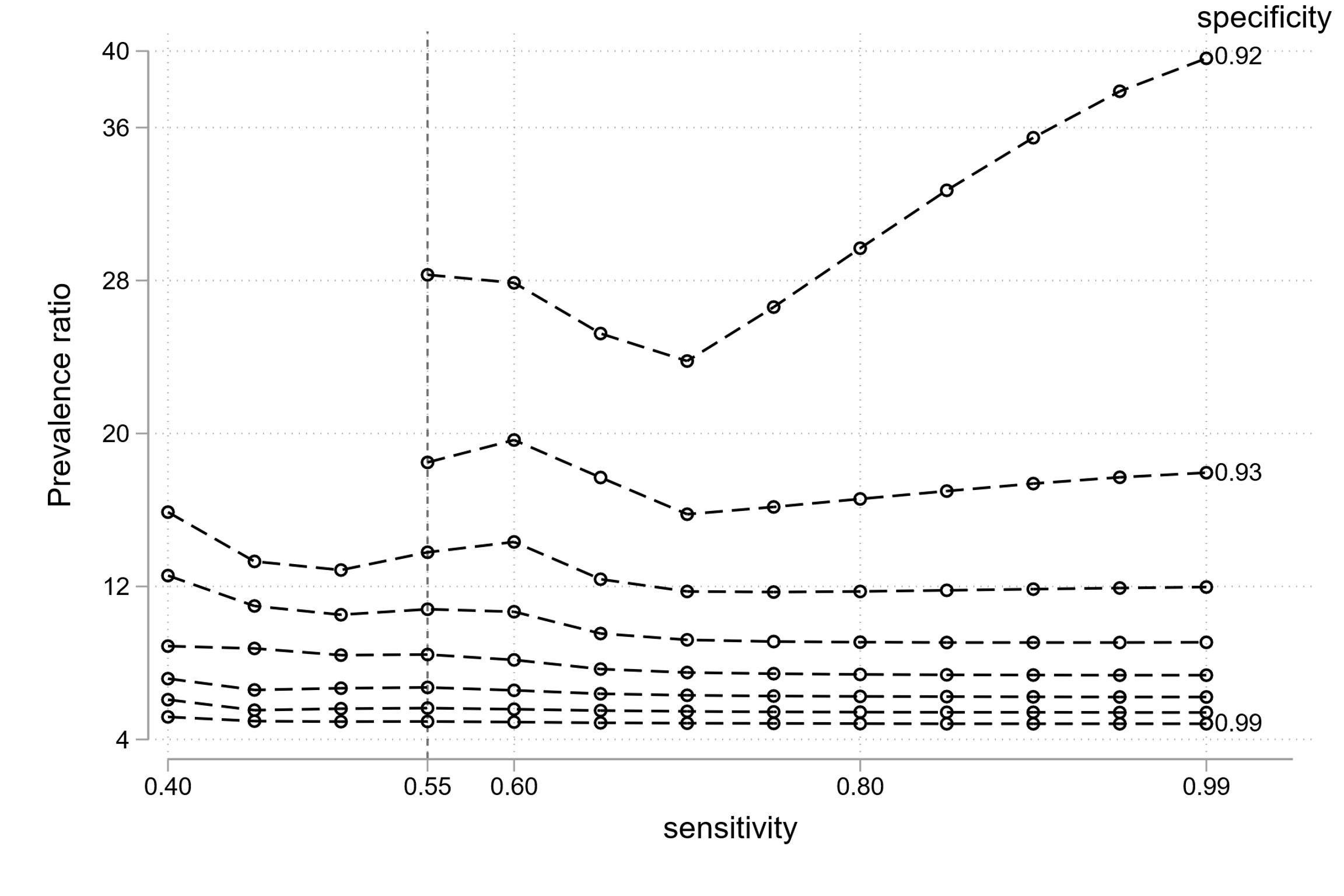


When the sensitivity≤0·55 and specificity ≤0·93, the models did not converge, shown as missing values in the graph.

| **S1 Table: Prevalence ratios for suspected OPMDs among participants recruited in an mHealth-based oral cancer screening study in Pune, India when sex is included as a covariate** | | |
| --- | --- | --- |
|  | **Sex excluded as a covariate** | **Sex included as a covariate** |
|  | **Adjusted**  **Prevalence Ratio**  **(95% CI)** | **Adjusted**  **Prevalence Ratio**  **(95% CI)** |
| **Model 1: Total study population (n=1234)** | | |
| **HIV status**  Uninfected  Living with HIV | *-*  ***1***·***47 (1***·***11 – 1***·***96)*** | *-*  ***1***·***47 (1***·***10 – 1***·***93)*** |
| **Model 2: People living with HIV (n=601)** | | |
| **Smokeless tobacco use**  Never  Former  Current | Ref  2·04 (0·96 – 4·34)  ***4***·***63 (3***·***06 – 7***·***01)*** | Ref  ***2***·***20 (1***·***03 – 4***·***69)***  ***4***·***92 (3***·***26 – 7***·***42)*** |
| **Sex**  Male  Female | **-** | Ref  ***1***·***64 (1***·***02 – 2***·***62)*** |
| **Smoked tobacco use**  Never  Former  Current | Ref  0·88 (0·56 – 1·39)  1·08 (0·61 – 1·91) | Ref  0·96 (0·60 – 1·55)  1·21 (0·67 – 2·18) |
| **Alcohol use**  Never  Former  Current | Ref  0·85 (0·54 – 1·36)  0·95 (0·66 – 1·37) | Ref  1·08 (0·64 – 1·82)  1·22 (0·77 – 1·93) |
| **Age/5 (years)** | ***1***·***10 (1***·***01 – 1***·***21)*** | ***1***·***12 (1***·***02 – 1***·***24)*** |
| **Recent CD4/ 50 (cells/mm^3^)** | 1·01 (0·98 – 1·04) | 1·01 (0·97 – 1·04) |
| **^a^** Model 1 was adjusted for age, sex, smoked tobacco use, smokeless tobacco use and alcohol use. The same variables were adjusted for in Model 2 (except for HIV status). CD4 was an additional adjustment variable included in Model 2. | | |

| **S2 Table: Adjusted prevalence ratios for suspected OPMDs among participants recruited in an mHealth-based oral cancer screening study in Pune, India comparing models where Oral HPV status was excluded as a covariate to models where Oral HPV values were imputed** | | |
| --- | --- | --- |
|  | **Oral HPV excluded as a covariate** | **Oral HPV values imputed** |
|  | **Adjusted**  **Prevalence Ratio**  **(95% CI)** | **Adjusted**  **Prevalence Ratio**  **(95% CI)** |
| **Model 1: Total study population** | | |
| **HIV status**  Uninfected  Living with HIV | *-*  ***1***·***47 (1***·***11 – 1***·***96)*** | Ref  ***1***·***48 (1***·***09 – 2***·***04)*** |
| **Human Papilloma Virus**  Negative  Positive | - | Ref  0·77 (0·37 – 1·63) |
| **Model 2: People living with HIV** | | |
| **Smokeless tobacco use**  Never  Former  Current | Ref  2·04 (0·96 – 4·34)  ***4***·***63 (3***·***06 – 7***·***01)*** | Ref  2·13 (0·97 – 4·68)  ***4***·***55 (2***·***86 – 7***·***23)*** |
| **Smoked tobacco use**  Never  Former  Current | Ref  0·88 (0·56 – 1·39)  1·08 (0·61 – 1·91) | Ref  0·93 (0·52 – 1·63)  1·10 (0·56 – 2·14) |
| **Alcohol use**  Never  Former  Current | Ref  0·85 (0·54 – 1·36)  0·95 (0·66 – 1·37) | Ref  0·86 (0·50 – 1·47)  0·96 (0·60 – 1·54) |
| **Age/5 (years)** | ***1***·***10 (1***·***01 – 1***·***21)*** | ***1***·***12 (1***·***01 – 1***·***24)*** |
| **Recent CD4/ 50 (cells/mm^3^)** | 1·01 (0·98 – 1·04) | 1·01 (0·98 – 1·05) |
| **Human Papilloma Virus**  Negative  Positive | - | Ref  0·79 (0·34 – 1·82) |
| Imputation technique: multiple imputation by chained equations (MICE)  Number of imputed datasets used: 25  There were 230 missing HPV values for Model 1 which were imputed  There were 8 missing CD4 values and 19 missing values for HPV in Model 2, both were imputed | | |

**S3 Fig**: Adjusted prevalence ratios for suspected OPMDs among current smokeless tobacco users by HIV status and duration of use, **when imputed oral HPV status is included as a covariate**


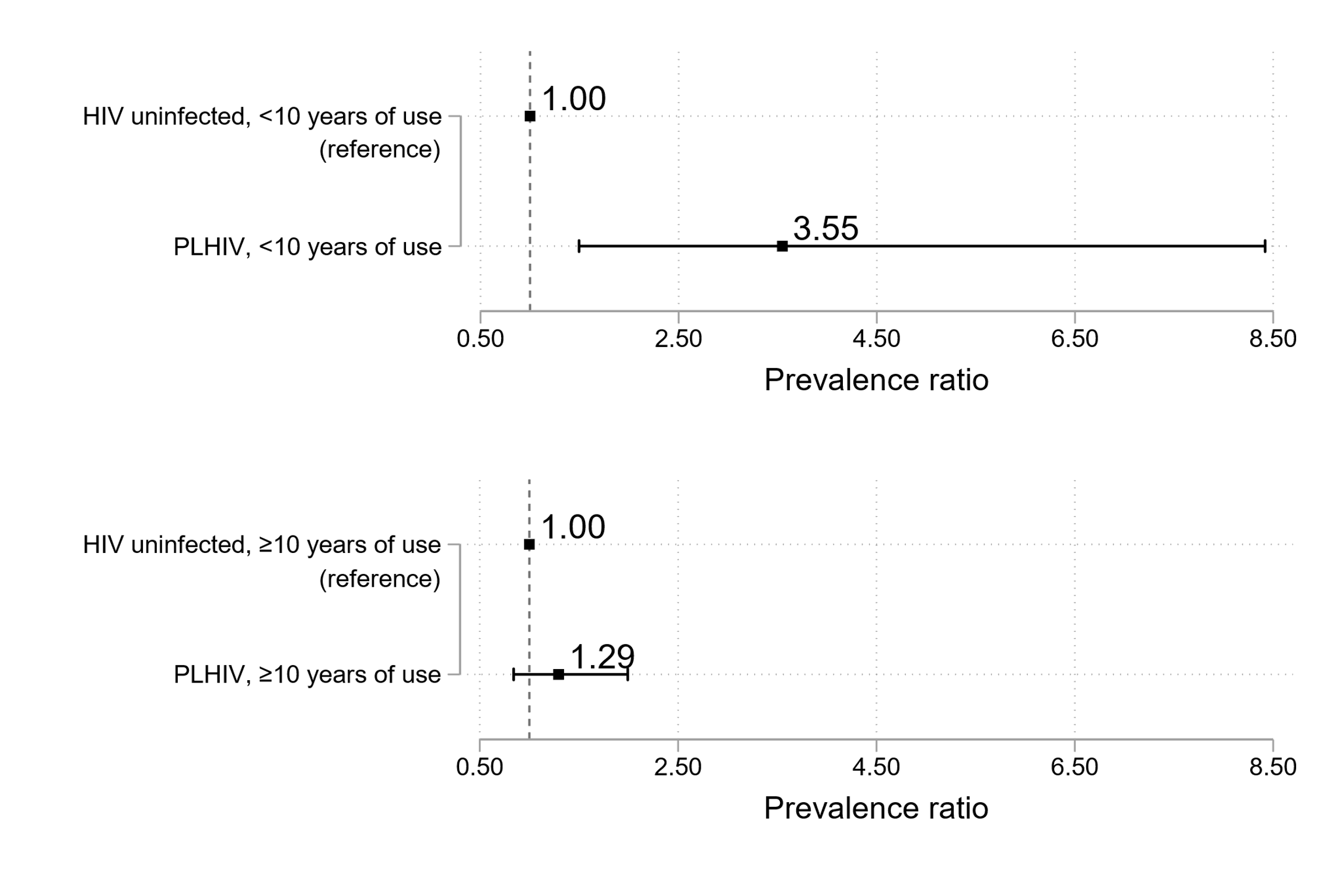

Supplement: S2 File — (DOCX) [file pone.0270876.s002.docx]
